# Reports of Cases Occurring in the Medical Ward of the Buffalo Hospital

**Published:** 1858-07

**Authors:** A. W. Nichols

**Affiliations:** Assistant to Chair of Clinical Medicine, University of Buffalo


ART. IV.—*Report of the Cases treated in the Male Medical Ward of the Buffalo Hospital of the Sisters of Charity,* AUSTIN FLINT, M. D. *Attending Physician, during the College Session of* 1857–8, *viz., from October* 11*th*, 1857, *to February* 24*th*, 1858. Reported by A. W. NICHOLS, M. D., Assistant to Chair of Clinical Medicine, University of Buffalo.

During the past year, the health of the city, and of the neighboring country, has been unusually good. No epidemic diseases have prevailed amongst the adult population since the beginning of last summer. The past fall and the earlier months of the present winter, were noted for unusual mildness and an equable temperature. The state of the financial world made it necessary for the poorer classes, who commonly resort to this Institution for numerous diseases not sufficiently serious to compel them to keep the bed, to continue their accustomed avocations. Much medical relief, also, has been extended gratuitously to the sick in this condition of life, by the physicians in the city and surrounding country. These causes have combined to render the Ward not as crowded as heretofore, at this season.

Although the foregoing circumstances have prevented many persons from entering the Hospital, yet no less than *thirty-five* different forms of disease have been under treatment, comprising as many as *seventy-four* cases. The number of patients presented to the notice of the class during the session, has been sixty-nine; the number treated in the Ward, has been sixty-three.

*Thirteen* of these sixty-three patients, have been attacked with other diseases during, or after the course of the particular affection for which they were admitted. Seven of the thirteen were restored to perfect health; in two instances, the principal disease exhibited a marked improvement; in two others, the chief disease remains stationary; and two patients succumbed to the disease. In one of these fatal cases, the patient was affected with valvular disease of the heart located at the mitral orifice: he presented many of the symptoms of typhoid fever, but the post-mortem examination revealed no lesions of the intestinal tube. In the other fatal case, the patient entered with sub-acute rheumatism; but soon the symptoms of *acute phthisis* appeared; and in a few weeks, death occurred.

The remaining eleven patients were attacked with the following diseases, respectively: the *first*, pulmonary tuberculosis and sub-acute rheumatism; the *second*, pulmonary tuberculosis and intermittent fever; the *third*, intermittent fever, pneumonitis, and erysipelas; the *fourth*, double pneumonitis and ecthyma; the *fifth*, remittent fever, pneumonitis, bronchitis, erysipelas, and prurigo; the *sixth*, intermittent fever, typhoid fever, and intercostal neuralgia; the *seventh*, intermittent fever and intercostal neuralgia; the *eighth*, ephemeral fever and intermittent fever; the *ninth*, erysipelas and chronic bronchitis; the *tenth*, diabetes mellitus and pulmonary tuberculosis; the *eleventh*, hemiplegia and prurigo.

Of the remaining fifty patients who had but a single disease, recovery took place in 17 instances; in 13, there was considerable improvement; in 6, the disease was stationary; in 7, the disease was slowly progressive; and 7 cases terminated fatally.

The nature of the disease in these seven fatal cases, was as follows: In

*one*, pulmonary tuberculosis; in a *second*, Morbus Brightii; in a *third*, abscess of the kidney; in a *fourth*, delirium tremens; in a *fifth*, Cerebral disease; in a *sixth*, typhoid fever; and in a *seventh*, the patient presented many of the symptoms of typhoid fever, but no lesions were discovered in the intestinal canal, at the post-mortem examination.

The diseases have embraced a wide range: there have been presented before the class, *eighty* different cases of disease; *six* of these were not under treatment in the Ward. In four of these six cases, there existed incipient tuberculosis; in one, valvular disease of the heart; and in another, albuminuria. Reference to the table will show the different forms of disease that have been under treatment, and the results.

A number of very interesting cases have been presented to the notice of the class; such as, *Acute Phthisis*, *Bright's Disease of the Kidney*, *Diabetes Mellitus*, *Fatty Substitution affecting the Heart*, *Abscess of the Kidney*, *Cerebral Disease*, *Paralysis with various modifications of sensibility.*

Thirty-two clinical lectures have been delivered by Prof. Flint, during the past session. Particular attention has been devoted to the physical signs, the methods of diagnosis, and the pathology of the various diseases.

The following subjects have been considered in all their bearings: *Pulmonary Tuberculosis*, *Acute Phthisis*, *Pneumonitis*, *Bronchitis*, *Valvular Disease of the Heart*, *Fatty Substitution affecting the Heart*, *Albuminuria and Bright's Disease of the Kidney*, *Diabetes Mellitus*, *Abscess of the Kidney*, *Pyelitis*, *Dysentery*, *Erysipelas*, *various forms of Skin Diseases*, *Sciatica*, *Gastralgia*, *Enteralgia*, *Intercostal Neuralgia*, *Spinal Affection*, *Hemiplegia and Paraplegia*, *Chronic Meningitis*, *Rheumatism*, *Intermittent*, *Remittent*, *and Typhoid Fevers.*

The following subjects have received attention, during the consideration of the diseases in which they formed a part: in *Pulmonary Tuberculosis*, the occurrence of dry pleuritis, of laryngitis, of hæmoptysis, of diarrhœa, of bulbous fingers and toes, of the broncho-cavernous respiration, of the treatment by alcoholic liquors and its results; in *Acute Phthisis*, the method of determining the disease and of distinguishing it from Bronchitis or from Typhoid fever; in *Pneumonitis*, the effect of co-existing skin disease, the reasons for a sustaining course of treatment in many cases; in *Valvular Disease of the Heart*, the method of locating the bellows-murmur; in *Dysentery*, the plan of treatment by saline laxatives and opiates; in *Diabetes Mellitus* and in *Albuminuria*, the methods of determining the presence of various substances in the urine; in *Paralysis*, the condition of the mental faculties after apoplexy succeeded by hemiplegia; in *Rheumatism*, the effects of various remedies, more particularly of the Iodide of Potassium and of the Wine of Colchicum, &c., &c.

| NAME OF THE DISEASE. | Number. | Recovered. | Improved. | Stationary. | Slowly Progres've. | Died. |
|---|---|---|---|---|---|---|
| Fever, Intermittent, | 10 | 10 | | | | |
| " Remittent, | 1 | 1 | | | | |
| " Typhoid, | 4 | 3 | | | | 1 |
| Rheumatism, | 7 | | | | | |
| Sub-acute, | 5 | 1 | 3 | 1 | | |
| Chronic, | 2 | | | 2 | | |
| Sciatica, | 1 | | 1 | | | |
| Gastralgia, | 2 | | 2 | | | |
| Enteralgia, | 2 | | 2 | | | |
| Intercostal Neuralgia, | 4 | 3 | 1 | | | |
| Spinal Affection, | 1 | | 1 | | | |
| Hemiplegia, | 2 | | 1 | 1 | | |
| Paraplegia, | 2 | | 1 | 1 | | |
| Cerebral Disease, | 1 | | | | | 1 |
| Delirium Tremens, | 2 | 1 | | | | 1 |
| Prurigo, | 2 | | | 2 | | |
| Herpes, | 1 | 1 | | | | |
| Ecthyma, | 2 | 2 | | | | |
| Psoriasis, | 2 | | 1 | | | |
| Erysipelas, | 3 | 3 | | | | |
| Albuminuria, Morbus Brightii, | 2 | | | | 1 | 1 |
| Diabetes Mellitus, | 1 | | | | 1 | |
| Abscess of the Kidney, | 1 | | | | | 1 |
| Diarrhœa, | 1 | 1 | | | | |
| Dysentery, Chronic, | 1 | | 1 | | | |
| Icterus, | 1 | 1 | | | | |
| Valvular Disease of Heart, | 1 | | | | | 1* |
| Fatty Substitution of do., | 1 | | | 1 | | |
| Pulmonary Tuberculosis, | 9 | | 1 | 2 | 5 | 1 |
| Acute Phthisis, | 1 | | | | | 1 |
| Pneumonitis, | 3 | 3 | | | | |
| Bronchitis, | 3 | | | | | |
| Acute, | 2 | 2 | | | | |
| Chronic, | 1 | | 1 | | | |
| Scrofula, | 1 | | 1 | | | |
| Unknown, | 2 | 2 | | | | |
| Symptoms of Typhoid Fever, | 2 | | | | | 2 |
| Total, | 78 | 34 | 17 | 10 | 7 | 9 |

* Included in those who died with the symptoms of Typhoid Fever; the valvular disease was not the cause of death.

